# From Unassuming to Unbelievable: A Case Report of Pyoderma Gangrenosum

**DOI:** 10.7759/cureus.53491

**Published:** 2024-02-03

**Authors:** Malina Mohtadi, Henry Alocha, Anas Mahmoud, Carlos Perez, Carl Lovaas

**Affiliations:** 1 Internal Medicine, St. Joseph's Regional Medical Center, Paterson, USA; 2 Internal Medicine, St. Joseph's University Medical Center, Paterson, USA; 3 Internal Medicine, St. Joseph’s University Medical Center, Paterson, USA; 4 Medicine, St. George's University Medical School, True Blue, GRD

**Keywords:** dermatology, autoimmune, inflammation, pyoderma gangrenosum (pg), pyoderma gangrenous

## Abstract

Pyoderma gangrenosum (PG) is an autoinflammatory skin disease, and there is no definitive test or established criterion for its diagnosis yet. This report discusses a case of a 34-year-old male patient who presented with an unassuming lesion that quickly worsened with physical manipulation. He was eventually diagnosed with PG. This report highlights the importance of a quick and accurate diagnosis of PG to prevent the worsening of a PG wound and its associated morbidity. It provides a detailed description of the condition accompanied by images to further spread awareness of this rare diagnosis.

## Introduction

Pyoderma gangrenosum (PG) is an autoinflammatory skin disease mediated by an altered neutrophilic response characterized by an increased presence in the skin and soft tissue. It is thought to result from an abnormal immune response comprising exaggerated neutrophil response, mutagenic changes, and dysregulation of the innate immune system. Studies from different parts of the world differ on the exact incidence of PG; however, American researchers determine it to be 5.8-20/100,000 with twice as many females affected as males [1,2,3]. Interestingly, the etiology spans a wide spectrum of causes, with 49% developing PG spontaneously, 27% following minor trauma, and 17% following surgery. Additionally, PG is associated with systemic diseases and can manifest in as many as 50% of those with inflammatory bowel disease (IBD). As there is no definitive test for the diagnosis of PG, it is unfortunately misdiagnosed about 10% of the time [1]. In this case report, we aim to highlight the importance of prompt clinical recognition and appropriate diagnosis of PG, especially given that trauma to the lesion caused by diagnostic testing can often exacerbate the lesion, a phenomenon known as pathergy.

## Case presentation

**Figure 1:**
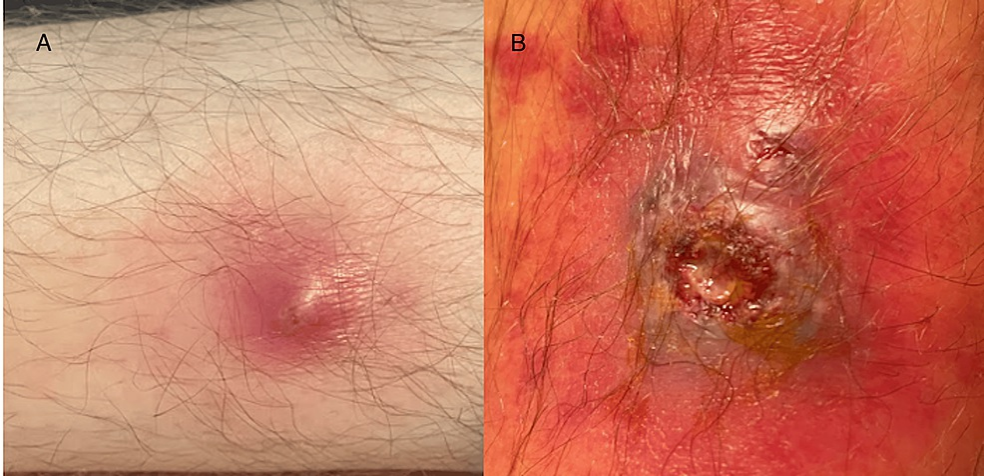
A: The lesion at the time of the original presentation before any physical manipulation. B: The lesion following incision and drainage

The patient was a 34-year-old male with no significant past medical history who presented with left lower extremity pain associated with a wound and surrounding erythema. He had first noticed the lesion 12 days ago measuring about 1.0 cm on his left anterior lower extremity. From the time of the initial presentation of the lesion (Figure 1A), the patient had multiple urgent care or emergency department (ED) visits, in which multiple wound cultures were obtained, and several trials of antibiotic regimens were given including cephalexin 500 mg for three days, bacitracin ointment for seven days, doxycycline 100 mg twice daily for seven days, and trimethoprim-sulfamethoxazole twice daily for seven days. he had also undergone a recent incision and drainage of the lesion (Figure 1B). During this period, the lesion had progressed from a small, red, pus-filled-appearing lesion to an open ulcerated lesion with a blue-colored border. The patient rated his pain as an 8/10 on the pain scale at the time of presentation.

In the ED, the lesion appeared to be very painful and distressing; the patient's pertinent lab values at that time are presented in Table 1.

**Table 1:** Significant laboratory values upon arrival at the emergency department

Labs	Patient value	Reference range
White blood cells	9.5 x 10^3^/mcL	4.5-11.0 x 10^3^/mcL
Erythrocyte sedimentation rate	8 mm/hr	0-10.0 mm/hr
C-reactive protein	20.7 mg/L	<9.9 mg/L

**Figure 2:**
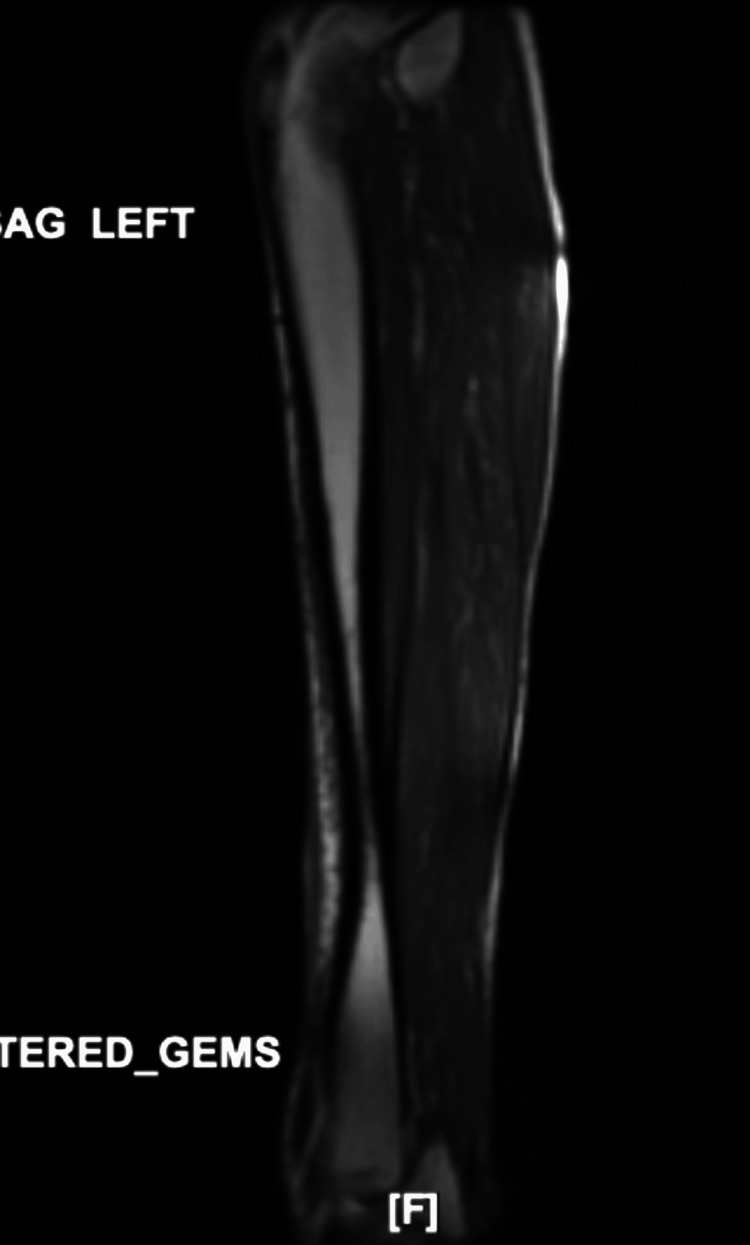
MRI of left tibia/fibula without contrast MRI: magnetic resonance imaging

An X-ray of the left tibia-fibula showed no fracture, or any evidence of osteomyelitis. Furthermore, an MRI of the left tibia-fibula indicated no evidence of a mass or collection, but showed nonspecific focal skin thickening, as an indication of possible cellulitis, as seen in Figure 2.

**Figure 3:**
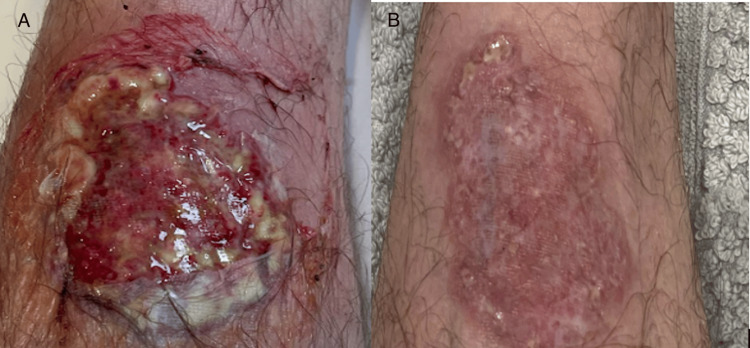
A: The lesion upon arrival to the emergency department. B: The lesion following treatment with steroid and cyclosporine

Simultaneously, both superficial and deep wound cultures were obtained, and the only significant finding was regarding the deep wound culture, which was positive for Candida lusitaniae. For further investigation, surgery was consulted, and collagenase Santyl® ointment was recommended for chemical debridement of the lesion, along with empiric methicillin-resistant Staphylococcus aureus (MRSA) coverage with vancomycin, and piperacillin/tazobactam. Unfortunately, the patient developed red man syndrome and was switched to ceftriaxone and linezolid. Given the significantly rapid progression of the lesion over 12 days following physical manipulation, negative wound cultures, and a wound swab showing minute fragments of mostly acute inflammatory cells, and squamous cells, PG was strongly suspected. This was further supported by the lack of other laboratory and constitutional signs of infection such as no leukocytosis, or fever. Thus, a biopsy of the lesion was deferred due to concerns for further trauma to the area, and oral prednisone was started. Following consultation with both dermatology and surgery, a diagnosis of PG was reached, and the patient was discharged with prednisone 60 mg daily and cyclosporine 100 mg twice a day, as well as instructions regarding appropriate outpatient consultations. Of note, improvement was noted in the patient's condition within one week of starting this treatment. Figure 3 shows the image of the lesion upon arrival at the ED (A) and following the above-mentioned treatment regimen (B).

## Discussion

PG is mediated by an abnormal autoinflammatory response. One study found that T-cells, such as clusters of differentiation (CD) 3+, CD163+, and macrophages, play a major role in inflammation as they have been found in the edges of ulcers in those with PG [4]. The subsequent cytokine signaling by these misplaced congregation of T-cells, like interleukin (IL) IL-8, IL-17, and IL-23, all play a role in the abnormal neutrophil response observed in these patients; IL-8 is a chemotactic mediator of neutrophils, IL-17 proliferates near the lesion itself, and IL-23 also activates neutrophils [4,5]. Thus, it is no surprise that the chemical storm that is inappropriately occurring, neutrophil chemotaxis to the area, leads to the pathognomonic picture that occurs with PG. This further supports the idea of its association with other neutrophil-predominant infiltrating diseases such as IBD, where neutrophils move to the intestinal tissue [6].

This inflammation is further exacerbated by the presence of IL-1β, which plays a role by binding and activating an inflammasome, a protein complex, that is normally activated by infection, and further brings proinflammatory chemo mediators to the area, and activates several proinflammatory mediators [6,7]. Variants in genes that code for the inflammasome complex also play a role in PG pathogenesis. Mutated variants of proline-serine-threonine phosphatase interacting protein 1 (PSTPIP1) increase the affinity of PSTPIP1 binding to pyrin, which subsequently leads to both the construction and hyperactivation of the inflammasome [7,8,9]. It is interesting to note that there are other pathways of inflammasome activation, aside from the association of IL-1β. A study performed in mice with a missense mutation of Ptpn6 reported neutrophilia and exudative inflammation. In this study, IL-1α is the key driving mediator in the autoinflammatory response by interaction with the inflammasome [7,10]. Thus, it was elicited that there are several paths of autoinflammatory activation than previously understood. As inflammatory processes occur, there is also an element of poor wound healing marked by the increase of matrix metalloproteinase (MMP), specifically MMP-9 and MMP-10 [11].

PG is classified into several types, the ulcerative presentation being the most common. Ulcerative PG typically presents as a painful papulopustular lesion, typically on the lateral lower extremity, which rapidly progresses into a necrotic ulcer with erythematous edges; often, the pain expressed by patients is far greater than what we would expect based on the appearance of the ulcer. Given the difficulty of diagnosing the condition, Su et al. have proposed that patients need to fulfill two major and two minor criteria, which are as follows - major criteria: rapid progression of a painful cutaneous ulcer with an irregular, violaceous border; exclusion of other causes of cutaneous ulceration; minor criteria: history suggestive of pathergy, history of systemic disease associated with PG, histopathological findings consistent with PD, and rapid response to treatment with steroids [1].

Although most patients with PG experience systemic symptoms, such as fever, malaise, and arthralgia, our patient did not have any of these symptoms [12]. Lesions are described as small papules, which can erode and quickly become necrotic [13]. This will lead clinicians to erroneously believe that they are infectious and implement surgical debridement, which can lead to rapid expansion of the lesion, up to 1 cm per day [13]. Patients may undergo multiple debridements, in the hopes of containing the lesion mistakenly attributed to a potentially infectious cause, only to make the condition worse, thereby making the diseased extremity susceptible to possible limb amputation [13]. Differentials to consider can also include compartment syndrome, as the textbook description of “pain out of proportion” can also be seen in PG [1]. Because of PG's association with autoimmune diseases, such as rheumatoid arthritis, irritable bowel disease, and hematological diseases, patients will typically be tested for other autoimmune titers and malignancies to ensure no further underlying conditions are present; however, in isolated PG, cultures and autoimmune workup will be negative [14,15]. 

Systemic corticosteroids (CS), at a dose of 0.5-1.0 mg/kg/day, are the first line of treatment for PG, and they lead to clinical improvement in 40-50% of patients [16]. CS works well against PG by mediating the transcription of NF-κB, and its effects therefore lead to the decrease in cytokines that play a role in inflammation [16]. Interestingly, randomized controlled trials have revealed that immunosuppressants such as cyclosporine, at a dose of 4 mg/kg/day, may be equally efficacious, with 47% of patients showing clinical improvement [7,16]. Cyclosporine, a calcineurin inhibitor, inhibits the synthesis of IL-2, which plays a role in T-cell activation [16]. Along with medical management therapy, appropriate wound care management also plays an important role. Tissue, Infection, Moisture, and Edge (TIME) represents an algorithm to follow for appropriate wound care in PG patients during the stages of healing. As described, a multifaceted approach is required to appropriately treat PG lesions.

## Conclusions

This case report highlights the importance of timely recognition and diagnosis of PG. In the process of diagnosing this condition, unnecessary procedures and physical manipulation of the lesion are often employed, which exacerbates the condition. Clinicians should have a thorough understanding of its presentation, which could aid in appropriate clinical decision-making when diagnosing PG, thereby minimizing the risk of aggravating the condition.
